# Supplementary material for: δ-catenin controls layer-specific transcriptional maturation of astrocytes via Zbtb20
Source: bioRxiv. 2026 May 13:2026.05.12.724361. Preprint. [Version 1] doi: 10.64898/2026.05.12.724361 (PMC13192610; doi:10.64898/2026.05.12.724361)
Supplement: 1 — Figure S1: Characterization of Ctnnd2-KO mouse model (related to Figure 1). Figure S2: Additional analysis and quality control of RNA sequencing (related to Figure 1). Figure S3: Additional analysis and quality control of snRNA sequencing (related to Figure 3). Figure S4: The effects of δ-catenin loss on glutamatergic neurons and oligodendroglia in the visual cortex (related to Figure 3). Figure S5: Spatial transcriptomic analysis of WT and Ctnnd2-KO visual cortex (related to Figure 4). Figure S6: Spatial transcriptomic analysis of oligodendrocytes (related to Figure 4). Figure S7: Quality control and additional multiomic CUT&RUN analyses (related to Figure 5). Figure S8: Quality control and additional astrocyte-specific CUT&RUN analyses (related to Figure 6). [file NIHPP2026.05.12.724361V1-supplement-1.pdf]

bioRxiv preprint doi: https://doi.org/10.64898/2026.05.12.724361; this version posted May 13, 2026. The copyright holder for this preprint (which was not certified by peer review) is the author/funder, who has granted bioRxiv a license to display the preprint in perpetuity. It is made available under a CC-BY 4.0 International license.

# SUPPLEMENTARY INFORMATION

## Supplementary Figures

**Figure S1:** Characterization of Ctnnd2-KO mouse model (related to Figure 1).

**Figure S2:** Additional analysis and quality control of RNA sequencing (related to Figure 1).

**Figure S3:** Additional analysis and quality control of snRNA sequencing (related to Figure 3).

**Figure S4:** The effects of δ-catenin loss on glutamatergic neurons and oligodendroglia in the visual cortex (related to Figure 3).

**Figure S5:** Spatial transcriptomic analysis of WT and Ctnnd2-KO visual cortex (related to Figure 4).

**Figure S6:** Spatial transcriptomic analysis of oligodendrocytes (related to Figure 4).

**Figure S7:** Quality control and additional multiomic CUT&RUN analyses (related to Figure 5).

**Figure S8:** Quality control and additional astrocyte-specific CUT&RUN analyses (related to Figure 6).

## Supplementary Tables

**Table S1**. Statistical analyses for main and supplemental figures, related to Figures 1-6 and S1-S8

**Table S2.** Differentially expressed genes from bulk RNAseq, related to Figure 1

**Table S3.** Differentially expressed genes from snRNAseq, related to Figures 3, 4, 5, and 6

**Table S4.** Astrocyte subcluster-defining genes from snRNAseq, related to Figures 3 and 4

**Table S5**. Custom Xenium panel information

**Table S6.** Astrocyte subcluster-defining genes from Xenium, related to Figure 4

**Table S7.** Differentially expressed genes from Xenium, related to Figure 4

**Table S8.** Homer enriched motifs, related to Figure 5

bioRxiv preprint doi: https://doi.org/10.64898/2026.05.12.724361; this version posted May 13, 2026. The copyright holder for this preprint (which was not certified by peer review) is the author/funder, who has granted bioRxiv a license to display the preprint in perpetuity. It is made available under a CC-BY 4.0 International license.

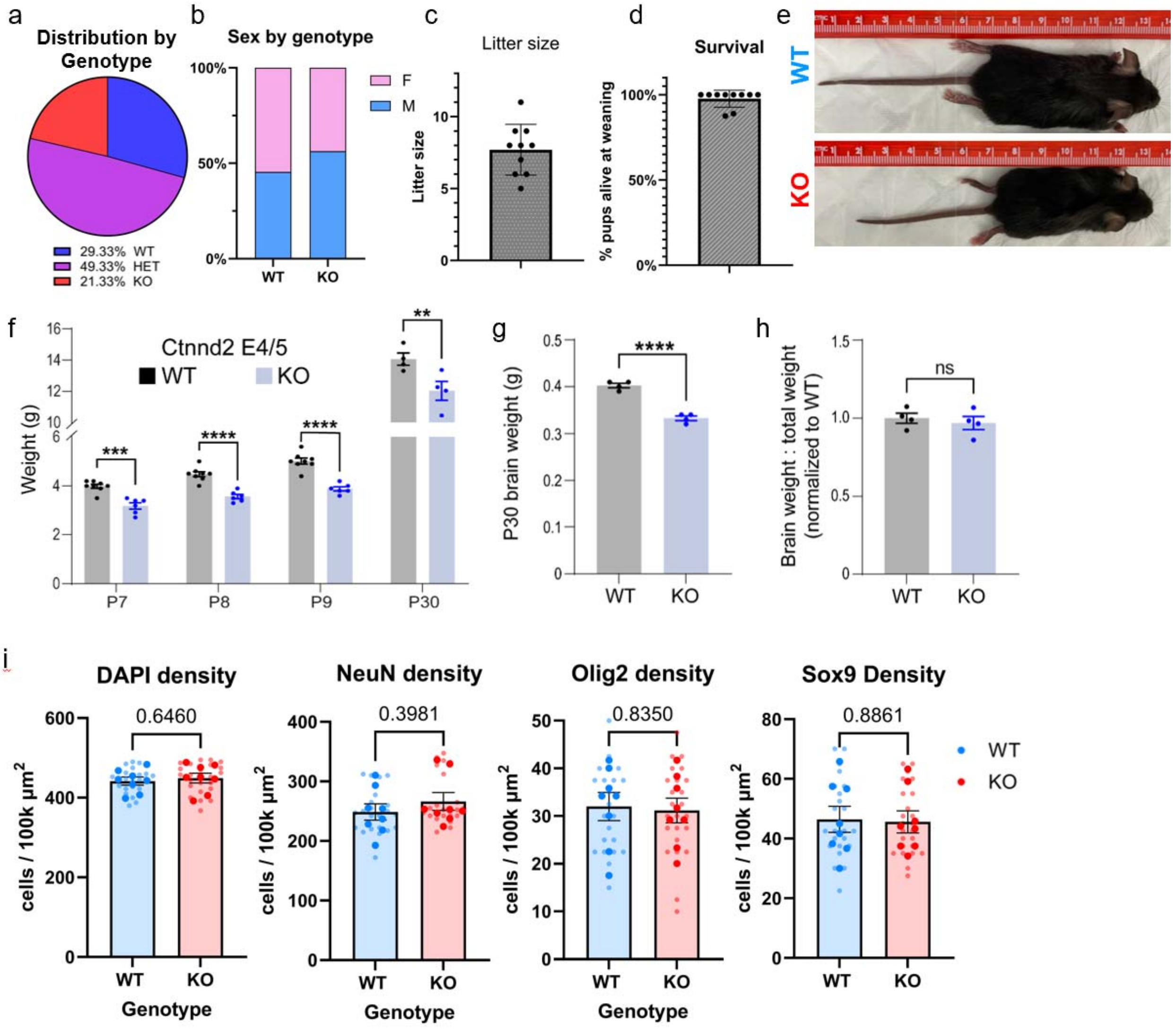


**Figure S1: Characterization of Ctnnd2-KO mouse model (related to Figure 1).** A-D) 10 independent litters from *Ctnnd2*-Het breeding pairs: (A) Genotype distribution, Chi-square test of independence, (2) = 0.9733, p = ns. B) Sex distribution, Chi-square test of independence, (1) = 0.4318, p = ns. C) Litter size and D) survival rate for offspring of *Ctnnd2*-Het breeding pairs. E) Photograph illustrating size difference between P30 Ctnnd2 E4/5 WT and KO sex-matched (male) littermates. F) Analysis of Ctnnd2 WT and KO body weight. N=4-8 littermate pairs per age. Data points represent mouse averages. Bars are mean ± s.e.m. Multiple unpaired two-tailed t-tests with Welch's correction. P7 (p=6.03e-4), P8 (p=1.8e-6), P9 (p=6e-6) and P30 (p=3.5e-2). N=4-8 animals per genotype per timepoint. G-H) Analysis of P30 *Ctnnd2* WT and KO brain weight (G) and brain weight normalized to body weight (H). N=4 sex-matched littermates, Welch's t-test. Brain weight (p=6e-4), normalized brain weight (ns). I) Unbinned density analysis of nuclei (DAPI), neurons (NeuN+), astrocytes (Sox9+), and oligodendrocytes (Olig2+) in the P21 visual cortex. N=8 mice of either sex per genotype, n=3 technical replicates/mouse. Welch's unpaired t-test.

bioRxiv preprint doi: https://doi.org/10.64898/2026.05.12.724361; this version posted May 13, 2026. The copyright holder for this preprint (which was not certified by peer review) is the author/funder, who has granted bioRxiv a license to display the preprint in perpetuity. It is made available under a CC-BY 4.0 International license.

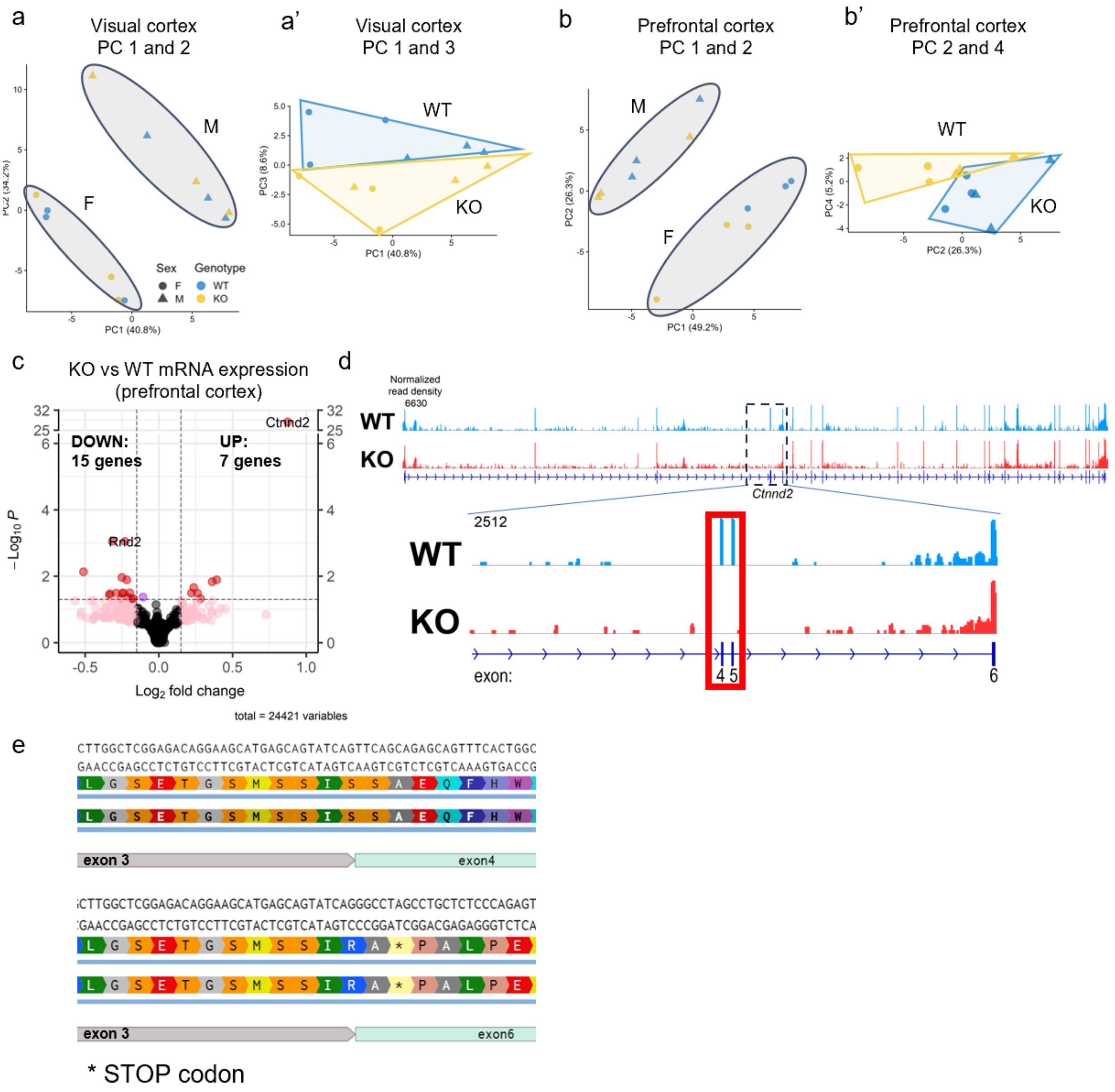


**Figure S2: Additional analysis and quality control of RNA sequencing (related to Figure 1).** A) PCA plots showing segregation of visual cortex samples by sex (A) and genotype (A'). B) PCA plots showing segregation of prefrontal cortex samples by sex (B) and genotype (B'). C) Volcano plot of DEGs in the prefrontal cortex of KO (N=6, 3 males, 3 females) compared to WT (N=6, 3 males, 3 females). Criteria for DEG selection: log2 fold change (FC) less than -0.15 or greater than 0.15; false discovery rate (FDR) < 0.05 as calculated by DESeq2 with Benjamini–Hochberg's correction. D) RNA sequencing reads aligned to *Ctnnd2* genomic DNA sequence. Reads aligned to exons 4 and 5 are absent in KO samples (red rectangle). E) Protein translation of KO allele showing early stop codon with excision of C*tnnd2* exons 4 and 5.

bioRxiv preprint doi: https://doi.org/10.64898/2026.05.12.724361; this version posted May 13, 2026. The copyright holder for this preprint (which was not certified by peer review) is the author/funder, who has granted bioRxiv a license to display the preprint in perpetuity. It is made available under a CC-BY 4.0 International license.

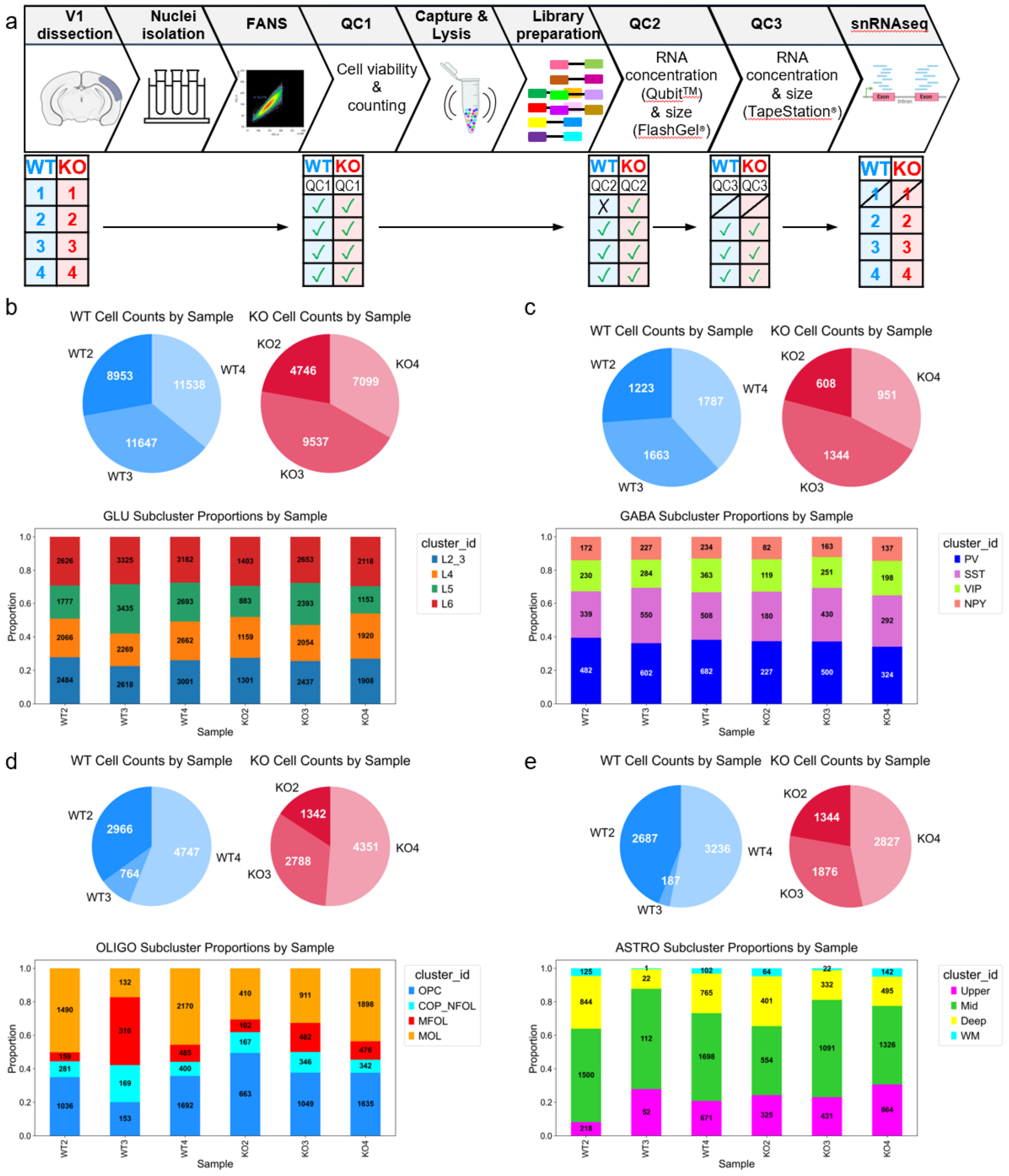


**Figure S3: Additional analysis and quality control of snRNA sequencing (related to Figure 3).** A) Detailed flow chart of snRNAseq experimental design, including sample filtering at successive quality control (QC) steps. B-E) Pie charts of sample distribution (top) and contingency plots (bottom) of cellular subtype distribution in each individual sample for glutamatergic neurons (B), GABAergic neurons (C), oligodendrocytes (D), and astrocytes (E).

bioRxiv preprint doi: https://doi.org/10.64898/2026.05.12.724361; this version posted May 13, 2026. The copyright holder for this preprint (which was not certified by peer review) is the author/funder, who has granted bioRxiv a license to display the preprint in perpetuity. It is made available under a CC-BY 4.0 International license.

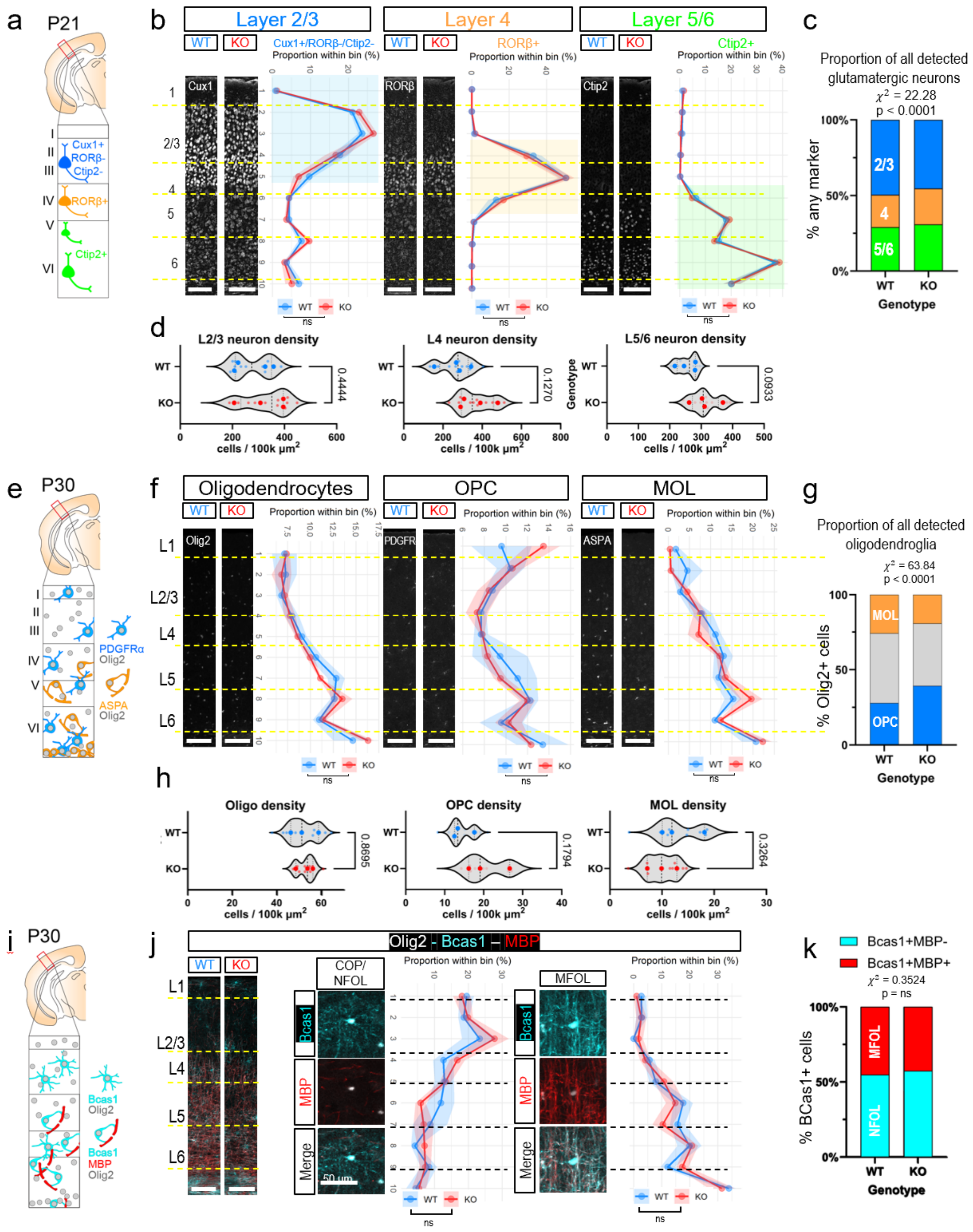

bioRxiv preprint doi: https://doi.org/10.64898/2026.05.12.724361; this version posted May 13, 2026. The copyright holder for this preprint (which was not certified by peer review) is the author/funder, who has granted bioRxiv a license to display the preprint in perpetuity. It is made available under a CC-BY 4.0 International license.

**Figure S4: The effects of δ-catenin loss on glutamatergic neurons and oligodendroglia in the visual cortex (related to Figure 3).** A) P21 visual cortex was stained for layer-specific markers (Cux1, RORβ, Ctip2) to identify glutamatergic neuron molecular subtypes and analyzed with respect to relative distance from the pia to corpus callosum. B) Representative images of Cux1 (left), RORβ (middle), and Ctip2 (right) staining and histograms of Layer 2/3 (Cux1+/RORβ-/Ctip2-), Layer 4 (RORβ+/Ctip2-), and Layer 5/6 (RORβ-/Ctip2+) molecular subtype distributions across V1 in WT and *Ctnnd2*-KO. No significant differences between genotypes (two-way ANOVA). Scale bars: 100 µm. All values represent the mean ± SEM. C) Barplot illustrating the proportion of L2/3, L4, and L5/6 neuron nuclei found in each genotype. Chi-square test of independence, $\chi^2$ (3) = 22.28, $p < 0.0001$. D) Density analysis of L2/3, L4, and L5/6 neuron nuclei within ROIs corresponding to L2/3, L4, and L5/6, respectively. Welch's unpaired t-test. For B-D: N=4 animals per genotype from 3 litters, n=2-3 technical replicates/mouse. E) P30 visual cortex was stained for stage-specific markers of oligodendrocyte lineage cells (Olig2, PDGFRα, ASPA) and analyzed with respect to relative distance from the pia to corpus callosum. OPC, Oligodendrocyte precursor cells; MOL, mature oligodendrocytes; NFOL, newly formed oligodendrocytes; MFOL, myelinating newly formed oligodendrocytes. F) Representative images of Olig2 (left), PDGFRα (middle), and ASPA (right) staining and histograms of oligodendrocytes (left), OPCs (middle), and MOLs (right) distribution across V1 in WT and *Ctnnd2*-KO. No significant differences between genotypes (two-way ANOVA). Scale bars: 100 µm. All values represent the mean ± SEM. G) Barplot illustrating the proportion of OPCs (PDGFRα+/ASPA-) and MOL (PDGFRα-/ASPA+) found in each genotype. Chi-square test of independence, $\chi^2$ (2) = 63.84, $p < 0.0001$. H) Unbinned density analysis of OPCs and MOLs in the P30 visual cortex. Welch's unpaired t-test. For F-H: N=3 animals per genotype from 2 litters, n=3 technical replicates/mouse. I) P30 visual cortex was stained for myelination status of newly formed oligodendrocytes (Bcas1, MBP) and analyzed with respect to relative distance from the pia to corpus callosum. J) Representative images of Bcas1 and MBP staining across V1 cortex (scale bars: 100 µm) with high magnification insets (scale bars: 50 µm) and histograms of NFOL (left), MFOL (right) distribution across V1 in WT and *Ctnnd2*-KO. No significant differences between genotypes (two-way ANOVA). All values represent the mean ± SEM. K) Barplot illustrating the proportion of NFOL (Bcas1+/MBP-) and MFOL (Bcas1+/MBP+) found in each genotype ($\chi^2$ (1) = 0.3524, p = ns). For J-K: N=4 animals per genotype from 4 litters, n=2-3 technical replicates/mouse.

bioRxiv preprint doi: https://doi.org/10.64898/2026.05.12.724361; this version posted May 13, 2026. The copyright holder for this preprint (which was not certified by peer review) is the author/funder, who has granted bioRxiv a license to display the preprint in perpetuity. It is made available under a CC-BY 4.0 International license.

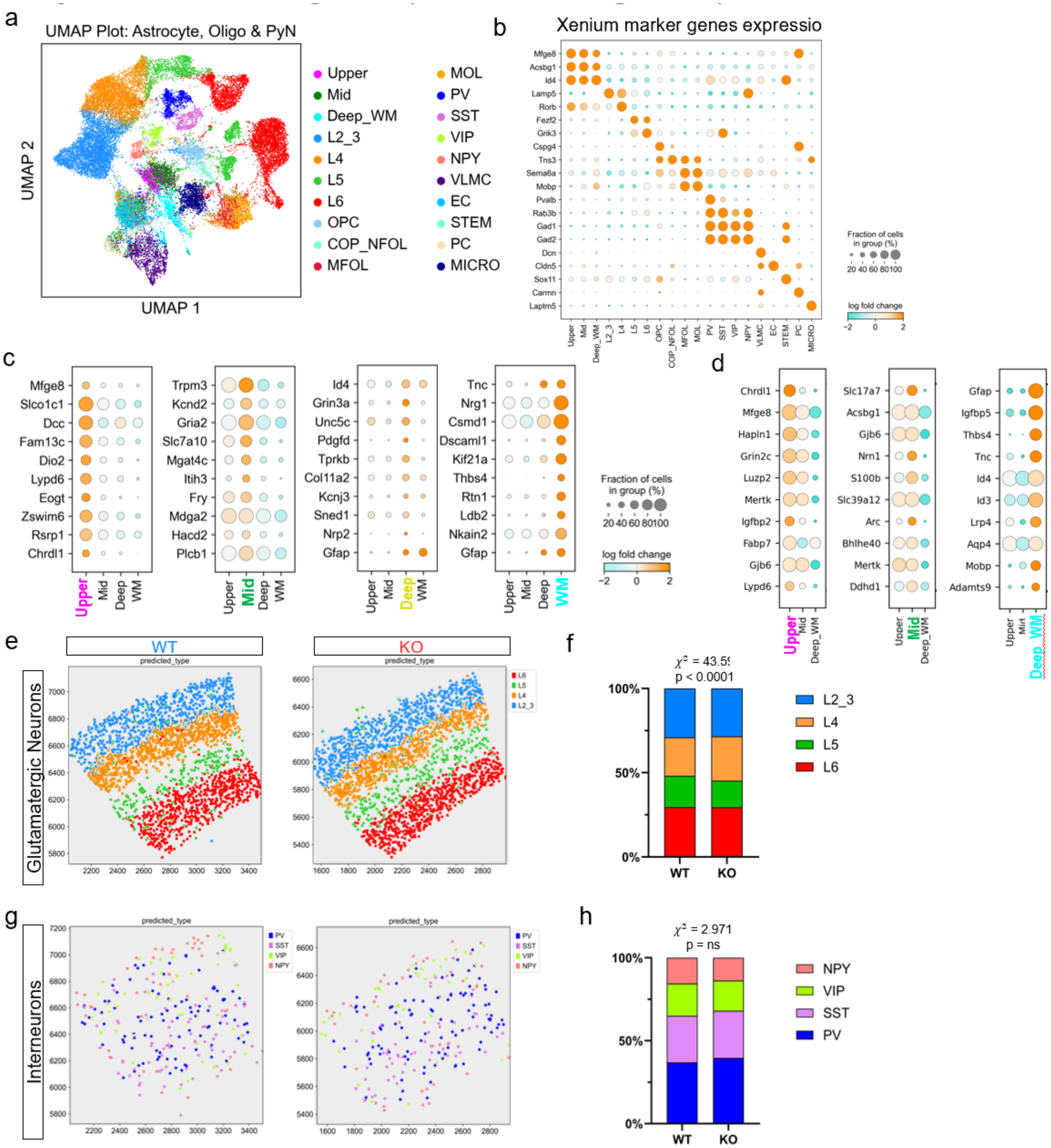


**Figure S5: Spatial transcriptomic analysis of WT and Ctnnd2-KO visual cortex (related to Figure 4).** A) UMAP V1 Xenium dataset, colored by cell identity. 31469 total cells detected (15222 WT, 16247 KO). B) Dotplot of marker gene expression across V1 cell types. C-D) Dotplots show cluster-defining genes for astrocyte subtypes from WT snRNAseq (C) and Xenium (D) data (Wilcoxon rank-sum test with Benjamini Hochberg correction comparing each cluster to every other cluster). Color of dot is log2 fold-change (log2FC), size of dot is fraction of cells expressing transcript. E-F) Spatial distribution (E) and proportion (F) of glutamatergic neuron subtypes in each genotype. Chi-square test of independence, (3) = 56.14 p < 0.0001. G-H) Spatial distribution (E) and proportion (F) of GABAergic interneuron subtypes in each genotype. Chi-square test of independence, (3) = 5.679 p = ns.

bioRxiv preprint doi: https://doi.org/10.64898/2026.05.12.724361; this version posted May 13, 2026. The copyright holder for this preprint (which was not certified by peer review) is the author/funder, who has granted bioRxiv a license to display the preprint in perpetuity. It is made available under a CC-BY 4.0 International license.

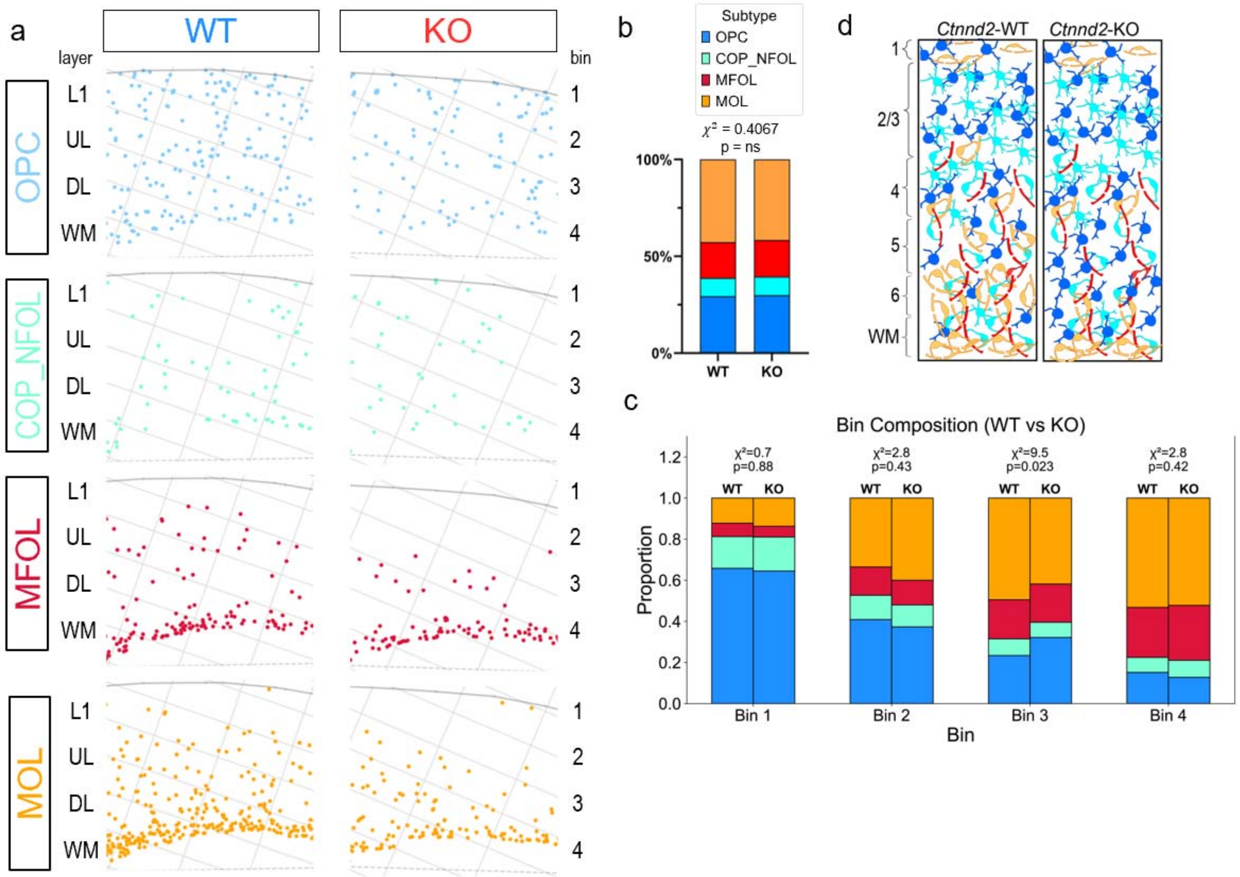


**Figure S6: Spatial transcriptomic analysis of oligodendrocytes (related to Figure 4).** A-B) Spatial distribution (A) and proportion (B) of oligodendrocyte lineage cell subtypes in each genotype. Chi-square test of independence, (3) = 0.4067, p = ns. C) Barplots show proportional contributions of each stage-specific subtype to the total oligodendroglia population within each of 4 equally sized cortical bins. D) Summary schematic of layer-specific changes in oligodendroglia subtypes.

bioRxiv preprint doi: https://doi.org/10.64898/2026.05.12.724361; this version posted May 13, 2026. The copyright holder for this preprint (which was not certified by peer review) is the author/funder, who has granted bioRxiv a license to display the preprint in perpetuity. It is made available under a CC-BY 4.0 International license.

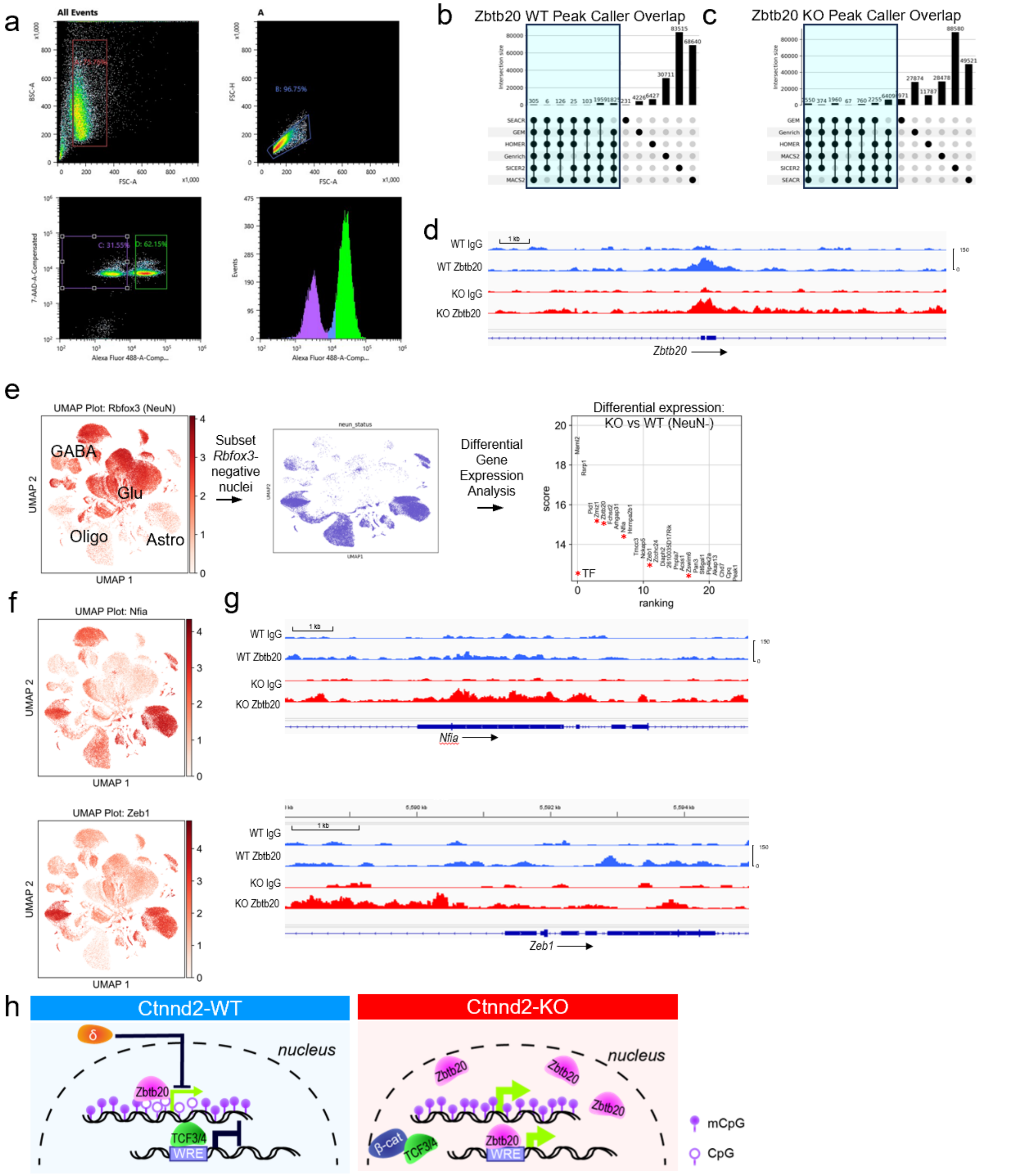


1807

1808

bioRxiv preprint doi: https://doi.org/10.64898/2026.05.12.724361; this version posted May 13, 2026. The copyright holder for this preprint (which was not certified by peer review) is the author/funder, who has granted bioRxiv a license to display the preprint in perpetuity. It is made available under a CC-BY 4.0 International license.

**Figure S7: Quality control and additional multiomic CUT&RUN analyses (related to Figure 5).** A) Fluorescence-activated nuclei sorting (FANS) gating strategy to isolate AF488-negative singlet glial nuclei from P28 cortex labeled by staining nuclei with AF488-conjugated NeuN antibody. Far left, debris exclusion gate; near left, singlet gate; near right, 7AAD+/AF488- nuclei gate; far right, AF488 histogram. B-C) UpSet plots showing overlap between the called peaks for WT (B) and KO (C) samples across all peak callers in the CARAS pipeline for Zbtb20 CUT&RUN. High confidence peak sets highlighted. D) Zbtb20 and IgG CUT&RUN read coverage at *Zbtb20* promoter. Merged tracks from N=4 WT and N=4 KO biological replicates. E) Bioinformatic pipeline for differential gene expression analysis of NeuN-negative nuclei. Asterisk indicates transcription factor or coactivator. F) UMAPs of all nuclei, annotated by expression of transcription regulator of interest. G) Zbtb20 and IgG CUT&RUN read coverage at *Nfia* (top) and *Zeb1* (bottom) promoters. Merged tracks from N=4 WT and N=4 KO biological replicates. H) Working model for cooperative transcriptional regulation by δ-catenin and Zbtb20.

bioRxiv preprint doi: https://doi.org/10.64898/2026.05.12.724361; this version posted May 13, 2026. The copyright holder for this preprint (which was not certified by peer review) is the author/funder, who has granted bioRxiv a license to display the preprint in perpetuity. It is made available under a CC-BY 4.0 International license.

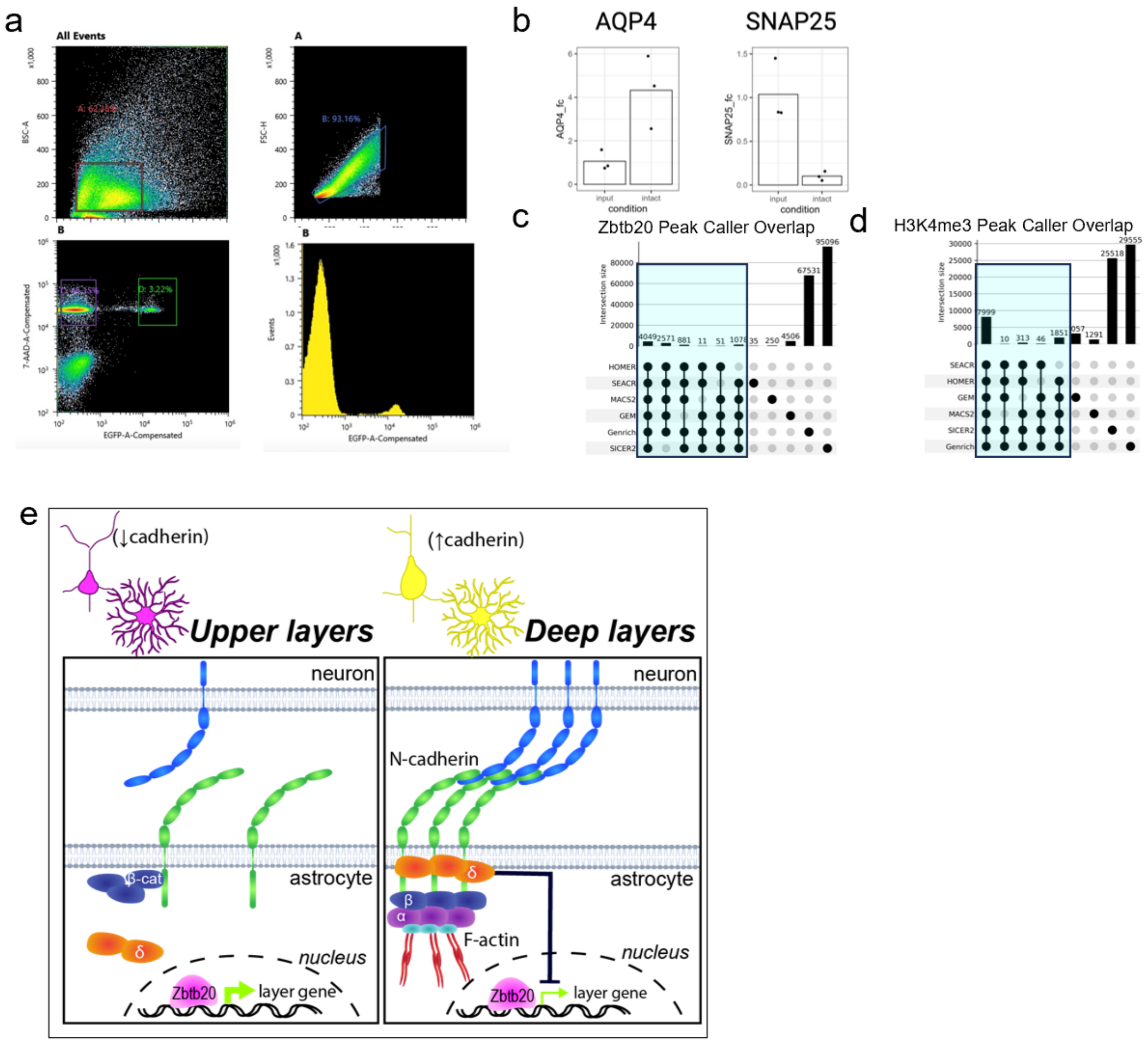


**Figure S8: Quality control and additional astrocyte-specific CUT&RUN analyses (related to Figure 6).** A) Fluorescence-activated nuclei sorting (FANS) gating strategy to isolate GFP+ singlet astrocyte nuclei from P21 cortex labeled by injecting CAG-Sun1/sfGFP mice with astrocyte-specific Cre AAV. Far left, debris exclusion gate; near left, singlet gate; near right, 7AAD+/GFP+ nuclei gate; far right, GFP histogram. B) Barplots showing mRNA expression of *Aqp4* and *Snap25* in GFP+ astrocyte nuclei isolated from p21 cortex by FANS. Values are fold-change compared to unsorted input nuclei. N=3 independent biological replicates.C-D) UpSet plots showing overlap between the called peaks for Zbtb20 (C) and H3K4me3 (D) CUT&RUN across all peak callers in the CARAS pipeline. E) Summary and working model for δ-catenin-dependent layer specificity of Zbtb20 transcriptional regulation in astrocytes.

bioRxiv preprint doi: https://doi.org/10.64898/2026.05.12.724361; this version posted May 13, 2026. The copyright holder for this preprint (which was not certified by peer review) is the author/funder, who has granted bioRxiv a license to display the preprint in perpetuity. It is made available under a CC-BY 4.0 International license.

**Table S1. Statistical analyses for main and supplemental figures, related to Figures 1-6 and S1-S8**

| Panel | Quantity | Test | P-value and N |
|---|---|---|---|
| 1G | Density of DAPI+ nuclei by cortical bin | 2-way ANOVA | N: 8 littermate pairs (4 WT-M, 4 WT-F, 3 KO-M, 5 KO-F)<br>P(bin): <2e-16; P(genotype): 0.5144; P(bin:genotype): 0.6976 |
| 1G | Density of NeuN+ nuclei by cortical bin | 2-way ANOVA | N: 8 littermate pairs (4 WT-M, 4 WT-F, 3 KO-M, 5 KO-F)<br>P(bin): 0.007065; P(genotype): 0.221239; P(bin:genotype): 0.937642 |
| 1G | Density of Sox9+ nuclei by cortical bin | 2-way ANOVA | N: 8 littermate pairs (4 WT-M, 4 WT-F, 3 KO-M, 5 KO-F)<br>P(bin): 8.272e-13; P(genotype): 0.7825; P(bin:genotype): 0.6566 |
| 1G | Density of Olig2+ nuclei by cortical bin | 2-way ANOVA | N: 8 littermate pairs (4 WT-M, 4 WT-F, 3 KO-M, 5 KO-F)<br>P(bin): 5.332e-13; P(genotype): 0.7913; P(bin:genotype): 0.2505 |
| 1I | VC RNA sequencing log2 fold-change of gene expression in KO compared to WT visual cortex | Wald test with Benjamini–Hochberg's correction | Criteria for DEG selection: log2 fold change (FC) less than -0.15 or greater than 0.15; false discovery rate (FDR) < 0.05 |
| 1J | Gene Ontology biological process category enrichment among upregulated genes | over-representation analysis using a hypergeometric distribution | Criteria for over-represented category: FDR < 0.05, q-value < 0.2 |
| 1K | Read count of upregulated genes in stemness-related GO categories | read count centered and scaled in the row direction | |
| 1L | Gene Ontology biological process category enrichment among downregulated genes | over-representation analysis using a hypergeometric distribution | Criteria for over-represented category: FDR < 0.05, q-value < 0.2 |
| 1M | Read count of downregulated genes in oxphos-related GO categories | read count centered and scaled in the row direction | |
| S1A | Genotype distribution of offspring in 10 independent litters from *Ctnnd2*-Het breeding pairs | Chi-square test of independence | N: 75 animals total (22 WT, 37 HET, 16 KO) |
| S1B | Sex distribution of offspring in 10 independent litters from *Ctnnd2*-Het breeding pairs | Chi-square test of independence | N: 38 animals total (10 WT-M, 12 WT-F, 9 KO-M, 7 KO-F) |
| S1F | Mouse body weight at P7, P8, P9, and P30 | Multiple unpaired two-tailed t-tests with Welch's correction | N: 4-8 animals per genotype per age (8 WT and 6 KO at P7, P8, and P9; 4 WT and 4 KO at P30)<br>P(P7):6.03e-4; P(P8): p=1.8e-6; P(P9): p=6e-6; |

bioRxiv preprint doi: https://doi.org/10.64898/2026.05.12.724361; this version posted May 13, 2026. The copyright holder for this preprint (which was not certified by peer review) is the author/funder, who has granted bioRxiv a license to display the preprint in perpetuity. It is made available under a CC-BY 4.0 International license.

| | | | |
|---|---|---|---|
| | | | P(P30): p=3.5e-2 |
| S1G | Mouse brain weight at P30 | Welch's t-test | N: 4 sex-matched littermate pairs<br>P: <0.0001 |
| S1H | Mouse brain weight normalized to body weight at P30 | Welch's t-test | N: 4 sex-matched littermate pairs<br>P: 0.5873 |
| S1I | Overall density of DAPI | Welch's t-test | N: 8 littermate pairs (4 WT-M, 4 WT-F, 3 KO-M, 5 KO-F)<br>P: 0.6460 |
| S1I | Overall density of NeuN | Welch's t-test | N: 8 littermate pairs (4 WT-M, 4 WT-F, 3 KO-M, 5 KO-F)<br>P: 0.3981 |
| S1I | Overall density of Olig2 | Welch's t-test | N: 8 littermate pairs (4 WT-M, 4 WT-F, 3 KO-M, 5 KO-F)<br>P: 0.8350 |
| S1I | Overall density of Sox9 | Welch's t-test | N: 8 littermate pairs (4 WT-M, 4 WT-F, 3 KO-M, 5 KO-F)<br>P: 0.8861 |
| S2C | PFC RNA sequencing log2 fold-change of gene expression in KO compared to WT visual cortex | Wald test with Benjamini–Hochberg's correction | Criteria for DEG selection: log2 fold change (FC) less than -0.15 or greater than 0.15; false discovery rate (FDR) < 0.05 |
| 2E | Width of binocular zone by condition and genotype | 2-way ANOVA with Tukey's multiple comparisons test | N: 7 NR Ctrl (6 WT, 1 Het), 6 MD Ctrl (2 WT, 4 Het), 5 NR KO, 6 MD KO<br>ANOVA: P(genotype): 0.6773; P(condition): <0.0001; P(interaction): 0.6530<br>Tukey: P(KO:NR vs. KO:MD): 0.0043; P(CTRL:NR vs. CTRL:MD): 0.0340 |
| 2G | Width of binocular zone by condition and genotype | 2-way ANOVA with Tukey's multiple comparisons test | N: 5 NR Ctrl (1 WT, 5 Het), 8 MD Ctrl (2 WT, 6 Het), 6 NR KO, 6 MD KO<br>ANOVA: P(genotype): 0.3523; P(condition): 0.0160; P(interaction): 0.1844<br>Tukey: P(KO:NR vs. KO:MD): 0.8107; P(CTRL:NR vs. CTRL:MD): 0.0481 |
| 3B | Unbiased Leiden clustering of nuclei in visual cortex from 3 WT and 3 KO animals | Leiden algorithm | N: 99966 nuclei total |
| 3C | snRNA sequencing log2 fold-change of gene expression in KO compared to WT glutamatergic nuclei | Wilcoxon rank-sum | N: 53340 nuclei (31958 WT, 21382 KO) |
| 3C | snRNA sequencing log2 fold-change of gene expression in KO compared to WT GABAergic nuclei | Wilcoxon rank-sum | N: 7576 nuclei (4673 WT, 2903 KO) |
| 3C | snRNA sequencing log2 fold-change of gene expression in KO | Wilcoxon rank-sum | N: 16958 nuclei 8477 WT, 8481 KO) |

bioRxiv preprint doi: https://doi.org/10.64898/2026.05.12.724361; this version posted May 13, 2026. The copyright holder for this preprint (which was not certified by peer review) is the author/funder, who has granted bioRxiv a license to display the preprint in perpetuity. It is made available under a CC-BY 4.0 International license.

| | | | |
|---|---|---|---|
| | compared to WT oligodendroglia nuclei | | |
| 3C | snRNA sequencing log2 fold-change of gene expression in KO compared to WT astrocyte nuclei | Wilcoxon rank-sum | N: 12157 nuclei 6110 WT, 6047 KO) |
| 3D | Gene Ontology biological process category enrichment among downregulated genes | over-representation analysis using a hypergeometric distribution | Criteria for over-represented category: FDR < 0.05, q-value < 0.2 |
| 3E | Subtype distribution of glutamatergic nuclei in visual cortex from 3 WT and 3 KO animals | Chi-square test of independence | N: 53340 nuclei (31958 WT, 21382 KO) |
| 3F | Subtype distribution of GABAergic nuclei in visual cortex from 3 WT and 3 KO animals | Chi-square test of independence | N: 7576 nuclei (4673 WT, 2903 KO) |
| 3G | Subtype distribution of oligodendroglia nuclei in visual cortex from 3 WT and 3 KO animals | Chi-square test of independence | N: 16958 nuclei (8477 WT, 8481 KO) |
| 3H | Subtype distribution of astrocyte nuclei in visual cortex from 3 WT and 3 KO animals | Chi-square test of independence | N: 12157 nuclei (6110 WT, 6047 KO) |
| S4B | Spatial frequency distribution of Cux1+/RORB-/Ctip2- nuclei | 2-way ANOVA | N: 4 littermate pairs (1 WT-M, 3 WT-F, 0 KO-M, 4 KO-F)<br>P(bin): <2e-16; P(genotype): 1.0000; P(bin:genotype): 0.3805 |
| S4B | Spatial frequency distribution of Cux1-/RORB+/Ctip2- and Cux1+/RORB+/Ctip2- nuclei | 2-way ANOVA | N: 4 littermate pairs (1 WT-M, 3 WT-F, 0 KO-M, 4 KO-F)<br>P(bin): <2e-16; P(genotype): 1.0000; P(bin:genotype): 0.9615 |
| S4B | Spatial frequency distribution of Cux1-/RORB-/Ctip2+ and Cux1+/RORB-/Ctip2+ nuclei | 2-way ANOVA | N: 4 littermate pairs (1 WT-M, 3 WT-F, 0 KO-M, 4 KO-F)<br>P(bin): <2e-16; P(genotype): 1.0000; P(bin:genotype): 0.9557 |
| S4C | Subtype distribution of glutamatergic nuclei in visual cortex from 4 WT and 4 KO animals | 2-way ANOVA | N: 12095 nuclei (5745 WT, 6350 KO)<br>Chi-square: 22.28, df: 2, P: <0.0001 |
| S4D | Density of Cux1+/RORB-/Ctip2- nuclei within L2/3 ROI | Welch's t-test | N: 4 littermate pairs (1 WT-M, 3 WT-F, 0 KO-M, 4 KO-F)<br>P: 0.4444 |
| S4D | Density of Cux1-/RORB+/Ctip2- and Cux1+/RORB+/Ctip2- within L4 ROI | Welch's t-test | N: 4 littermate pairs (1 WT-M, 3 WT-F, 0 KO-M, 4 KO-F)<br>P: 0.1270 |

bioRxiv preprint doi: https://doi.org/10.64898/2026.05.12.724361; this version posted May 13, 2026. The copyright holder for this preprint (which was not certified by peer review) is the author/funder, who has granted bioRxiv a license to display the preprint in perpetuity. It is made available under a CC-BY 4.0 International license.

| | | | |
|---|---|---|---|
| S4D | Density of Cux1-/RORB-/Ctip2+ and Cux1+/RORB-/Ctip2+ nuclei within L5/6 ROI | Welch's t-test | N: 4 littermate pairs (1 WT-M, 3 WT-F, 0 KO-M, 4 KO-F)<br>P: 0.0933 |
| S4F | Spatial frequency distribution of Olig2+ nuclei | 2-way ANOVA | N: 3 littermate pairs (1 WT-M, 2 WT-F, 2 KO-M, 1 KO-F)<br>P(bin): 6.526e-13; P(genotype): 1.0000; P(bin:genotype): 0.8088 |
| S4F | Spatial frequency distribution of Olig2+/Pdgfra+/Aspa- nuclei | 2-way ANOVA | N: 3 littermate pairs (1 WT-M, 2 WT-F, 2 KO-M, 1 KO-F)<br>P(bin): 0.003315; P(genotype): 1.000000; P(bin:genotype): 0.791523 |
| S4F | Spatial frequency distribution of Olig2+/Pdgfra-/Aspa+ nuclei | 2-way ANOVA | N: 3 littermate pairs (1 WT-M, 2 WT-F, 2 KO-M, 1 KO-F)<br>P(bin): 1.472e-13; P(genotype): 1.000; P(bin:genotype): 0.479 |
| S4G | Subtype distribution of oligodendrocyte nuclei in visual cortex from 3 WT and 3 KO animals | 2-way ANOVA | N: 4046 cells (2028 WT, 2018 KO)<br>Chi-square: 63.84, df: 2, P: <0.0001 |
| S4H | Overall density of Olig2+ nuclei | Welch's t-test | N: 3 littermate pairs (1 WT-M, 2 WT-F, 2 KO-M, 1 KO-F)<br>P: 0.8695 |
| S4H | Overall density of Olig2+/Pdgfra+/Aspa- nuclei | Welch's t-test | N: 3 littermate pairs (1 WT-M, 2 WT-F, 2 KO-M, 1 KO-F)<br>P: 0.1794 |
| S4H | Overall density of Olig2+/Pdgfra-/Aspa+ nuclei | Welch's t-test | N: 3 littermate pairs (1 WT-M, 2 WT-F, 2 KO-M, 1 KO-F)<br>P: 0.3264 |
| S4J | Spatial frequency distribution of Bcas1+/MBP- cells | 2-way ANOVA | N:6 littermate pairs (3 WT-M, 3 WT-F, 5 KO-M, 1 KO-F)<br>P(bin): 1.496e-12; P(genotype): 0.7947; P(bin:genotype): 0.7745 |
| S4J | Spatial frequency distribution of Bcas1+/MBP+ cells | 2-way ANOVA | N:6 littermate pairs (3 WT-M, 3 WT-F, 5 KO-M, 1 KO-F)<br>P(bin): <2e-16; P(genotype): 0.8083; P(bin:genotype): 0.7717 |
| S4K | Subtype distribution of Bcas1+ cells in visual cortex from 6 WT and 6 KO animals | Chi-square test of independence | N: 515 cells (251 WT, 264 KO)<br>Chi-square: 0.3524, df: 1, P: 0.5528 |
| 4D | Gene expression in *Ctnnd2*-KO compared to WT samples in V1 ROI | Wilcoxon rank-sum test with Benjamini Hochberg correction comparing KO to WT | 20 highest Wilcoxon scores |
| 4E | Gene expression in *Ctnnd2*-KO compared to WT samples in V1 ROI | Wilcoxon rank-sum test with Benjamini Hochberg correction comparing KO to WT | 20 lowest Wilcoxon scores |
| 4G | Subtype distribution of astrocytes in V1 ROI from 2 WT and 2 KO animals | Chi-square test of independence | N: 3117 nuclei total (1576 WT, 1541 KO)<br>Chi-square: 13.24, df: 2, P: 0.0013 |

bioRxiv preprint doi: https://doi.org/10.64898/2026.05.12.724361; this version posted May 13, 2026. The copyright holder for this preprint (which was not certified by peer review) is the author/funder, who has granted bioRxiv a license to display the preprint in perpetuity. It is made available under a CC-BY 4.0 International license.

| | | | |
|---|---|---|---|
| 4H | Subtype distribution of astrocytes in V1 bin 1 ROI from 2 WT and 2 KO animals | Chi-square test of independence | N: 1073 nuclei total (527 WT, 546 KO)<br>Chi-square: 11.8987, df: 2, P: 0.0026 |
| 4H | Subtype distribution of astrocytes in V1 bin 2 ROI from 2 WT and 2 KO animals | Chi-square test of independence | N: 688 nuclei total (343 WT, 345 KO)<br>Chi-square: 3.3452, df: 2, P: 0.1878 |
| 4H | Subtype distribution of astrocytes in V1 bin 3 ROI from 2 WT and 2 KO animals | Chi-square test of independence | N: 625 nuclei total (316 WT, 309 KO)<br>Chi-square: 11.9400, df: 2, P: 0.0026 |
| 4H | Subtype distribution of astrocytes in V1 bin 4 ROI from 2 WT and 2 KO animals | Chi-square test of independence | N: 727 nuclei total (388 WT, 339 KO)<br>Chi-square: 1.9180, df: 2, P: 0.38 |
| S5F | Subtype distribution of glutamatergic neurons in V1 ROI from 2 WT and 2 KO animals | Chi-square test of independence | N: 18264 nuclei total (8380 WT, 9884 KO)<br>Chi-square: 43.59, df: 3, P: <0.0001 |
| S5H | Subtype distribution of GABAergic neurons in V1 ROI from 2 WT and 2 KO animals | Chi-square test of independence | N: 3538 nuclei total (1850 WT, 1688 KO)<br>Chi-square: 2.971, df: 3, P: 0.3961 |
| S6B | Subtype distribution of oligodendroglia in V1 ROI from 2 WT and 2 KO animals | Chi-square test of independence | N: 3117 nuclei total (1576 WT, 1541 KO)<br>Chi-square: 0.4067, df: 3, P: 0.9388 |
| S6C | Subtype distribution of oligodendroglia in V1 bin 1 ROI from 2 WT and 2 KO animals | Chi-square test of independence | N: 526 nuclei total (278 WT, 248 KO)<br>Chi-square: 0.6876, df: 3, P: 0.8761 |
| S6C | Subtype distribution of oligodendroglia in V1 bin 2 ROI from 2 WT and 2 KO animals | Chi-square test of independence | N: 613 nuclei total (313 WT, 300 KO)<br>Chi-square: 2.7748, df: 3, P: 0.4277 |
| S6C | Subtype distribution of oligodendroglia in V1 bin 3 ROI from 2 WT and 2 KO animals | Chi-square test of independence | N: 914 nuclei total (479 WT, 435 KO)<br>Chi-square: 9.5061, df: 3, P: 0.02327 |
| S6C | Subtype distribution of oligodendroglia in V1 bin 4 ROI from 2 WT and 2 KO animals | Chi-square test of independence | N: 1481 nuclei total (778 WT, 703 KO)<br>Chi-square: 2.8181, df: 3, P: 0.4205 |
| 5E | Genomic feature distribution of high confidence peaks in 4 WT and 4 KO samples | Chi-square test of independence | N: 17724 loci (4349 WT 13375 KO)<br>Chi-square: 283.6, df: 6, P: <0.0001 |
| 5J | Motif enrichment among WT-only Zbtb20-bound promoters | over-representation analysis using a hypergeometric distribution | N: 2103 WT promoters, 17807 background promoters<br>Criteria for enriched motif: p-value < 0.05 |
| 5J | Motif enrichment among KO-only Zbtb20-bound promoters | over-representation analysis using a hypergeometric | N: 951 WT promoters, 18868 background promoters<br>Criteria for enriched motif: p-value < 0.05 |

bioRxiv preprint doi: https://doi.org/10.64898/2026.05.12.724361; this version posted May 13, 2026. The copyright holder for this preprint (which was not certified by peer review) is the author/funder, who has granted bioRxiv a license to display the preprint in perpetuity. It is made available under a CC-BY 4.0 International license.

| | | | |
|---|---|---|---|
| | | distribution | |
| 5K | DEG enrichment among genes with Zbtb20-bound promoters | Fisher's exact test | N: 35795 genes total (32227 overall, 3568 Zbtb20-bound) |
| 6E | Gene expression in *Ctnnd2*-KO compared to WT astrocyte nuclei | Wilcoxon rank-sum test with Benjamini Hochberg correction comparing KO to WT | |
| 6F | DEG enrichment among genes with layer-enriched expression in astrocytes | Fisher's exact test | N: 3343 genes total (471 DEG, 2872 non-DEG)<br>P: <0.0001 |
| 6I | Normalized mean fluorescent intensity of Tnc by cortical bin | 2-way ANOVA | N: 3 littermate pairs (2 WT-M, 1 WT-F, 2 KO-M, 1 KO-F)<br>P(bin): 9.157e-15; P(genotype): 0.1090; P(bin:genotype): 0.7546 |
| 6J | Normalized mean fluorescent intensity of Id4 by cortical bin | 2-way ANOVA | N: 3 littermate pairs (2 WT-M, 1 WT-F, 2 KO-M, 1 KO-F)<br>P(bin): 9.431e-16; P(genotype): 0.6188; P(bin:genotype): 0.7279 |
| 6K | Normalized mean fluorescent intensity of Mfge8 by cortical bin | 2-way ANOVA with Tukey's multiple comparisons test | N: 3 littermate pairs (2 WT-M, 1 WT-F, 2 KO-M, 1 KO-F)<br>ANOVA: P(bin): 2.462e-12; P(genotype): 0.022735; P(bin:genotype): 0.004651<br>Tukey: P(bin1): 0.0321; P(bin7): 0.0355; P(bin9): 0.0138; P(bin10): 0.0004 |
